# Eco-Friendly Octylsilane-Modified Amino-Functional Silicone Coatings for a Durable Hybrid Organic–Inorganic Water-Repellent Textile Finish

**DOI:** 10.3390/polym17111578

**Published:** 2025-06-05

**Authors:** Mariam Hadhri, Claudio Colleoni, Agnese D’Agostino, Mohamed Erhaim, Raphael Palucci Rosa, Giuseppe Rosace, Valentina Trovato

**Affiliations:** 1 Department of Engineering and Applied Sciences, University of Bergamo, 24044 Dalmine, BG, Italy; mariam.hadhri@unibg.it (M.H.); m.erhaim@studenti.unibg.it (M.E.); raphael.rosa@unibg.it (R.P.R.); giuseppe.rosace@unibg.it (G.R.); 2 Argochem Srl, 24044 Dalmine, BG, Italy; claudio.colleoni@argochem.eu; 3 Local CSGI (Inter-University Center for Colloid and Surface Science) Research Unit, 24044 Dalmine, BG, Italy; 4 Local INSTM (National Consortium of Materials Science and Technology) Research Unit, 24044 Dalmine, BG, Italy

**Keywords:** hybrid organic–inorganic coatings, sol–gel, water repellence, non-fluorinated water-repellent coating, eco-friendly finishes, cotton fabrics, polyester fabrics

## Abstract

The widespread phase-out of long-chain per- and poly-fluoroalkyl substances (PFASs) has created an urgent need for durable, fluorine-free water-repellent finishes that match the performance of legacy chemistries while minimising environmental impact. Here, the performance of an eco-friendly hybrid organic–inorganic treatment obtained by the in situ hydrolysis–condensation of triethoxy(octyl)silane (OS) in an amino-terminated polydimethylsiloxane (APT-PDMS) aqueous dispersion was investigated. The sol was applied to plain-weave cotton and polyester by a pad-dry-cure process and benchmarked against a commercial fluorinated finish. Morphology and chemistry were characterised by SEM–EDS, ATR-FTIR, and Raman spectroscopy; wettability was assessed by static contact angle, ISO 4920 spray ratings, and AATCC 193 water/alcohol repellence; and durability, handle, and breathability were evaluated through repeated laundering, bending stiffness, and water-vapour transmission rate measurements. The silica/PDMS coating formed a uniform, strongly adherent nanostructured layer conferring static contact angles of 130° on cotton and 145° on polyester. After five ISO 105-C10 wash cycles, the treated fabrics still displayed a spray rating of 5/5 and AATCC 193 grade 7, outperforming or equalling the fluorinated control, while causing ≤5% loss of water-vapour permeability and only a marginal increase in bending stiffness. These results demonstrate that the proposed one-step, water-borne sol–gel process affords a sustainable, industrially scalable route to high-performance, durable, water-repellent finishes for both natural and synthetic textiles, offering a viable alternative to PFAS-based chemistry for outdoor apparel and technical applications.

## 1. Introduction

Textiles are widely used materials that play a crucial role in various aspects of daily life [1]. Indeed, beyond clothing for protection or fashion, textiles are employed in the medical, military sports, and other technical sectors [2]. In recent decades, global fibre consumption has steadily increased, with per capita consumption rising from 8.3 kg in 1975 to 15.5 kg in 2023 [3]. This growth trend is confirmed by global fibre production, which has more than doubled since 2000, from 58 million tons to 124 million tons in 2023, 64% of which comes from the petrochemical industry [3].

Among them, polyester maintains its dominant position, accounting for 57% of total fibre production, while the remaining 36% of fibres consist of cotton (24%), artificial cellulosic fibres (6%), and other natural fibres (6%). Despite the prevalence of synthetic fibres, global cotton production is expected to grow at an annual rate of 1.6%, increasing from 126.5 million bales in 2022/2023 to an estimated 141.3 million bales in 2031/2032 [4]. Cotton remains one of the most widely used natural fibres in the textile industry, owing to its exceptional properties such as softness, breathability, moisture absorption, and comfort. Thus, polyester and cotton represent a significant share of the global textile fibre market, justifying their selection as representative substrates for evaluating innovative finishing technologies. Nonetheless, these two fibre types exhibit fundamentally distinct physicochemical structures and properties.

Conventional polyester fibres are synthesised through the condensation polymerisation of ethylene glycol and terephthalic acid, resulting in polyethylene terephthalate (PET). The molecular structure of PET includes several functional groups, such as ester bonds, aromatic phenyl rings, and terminal hydroxyl groups, with ester functionalities comprising approximately 46% of the total structure [4,5]. Chemically, cotton is composed predominantly of cellulose (90–94%) [5], a polysaccharide consisting of linear chains of β-D-pyran glucose units linked by glycosidic bonds, resulting in lightweight and soft fibres, offering comfort in various apparel applications [4]. Both fibres are inherently hydrophilic: cotton because of numerous hydroxyl groups along the cellulose chains, whereas polyester, although intrinsically hydrophobic, absorbs liquid by capillary action [6]. This characteristic can limit their application in the field of technical textiles. To address this limitation, the development of water-repellent treatments for fabrics has garnered considerable attention, aiming to enhance their performance across various applications while preserving their environmental advantages.

Several researchers have endeavoured to impart hydrophobic characteristics to polyester and cotton fabrics to address this limitation, primarily through three main strategies [7,8]: (i) modifying the surface by incorporating materials with inherently low surface energy; (ii) increasing surface roughness by introducing micro- and nanostructured features; and (iii) utilizing a synergistic combination of both methods. The correlation between surface roughness and hydrophobic behaviour has been extensively described by the theoretical frameworks proposed by Wenzel and Cassie-Baxter [8]. Specifically, the Wenzel model describes a wetting regime in which the liquid fully impregnates the rough surface, whereas the Cassie-Baxter model characterizes a state where air pockets are retained between the textured solid and the contacting liquid, thereby reducing the actual contact area [9]. Achieving hydrophobicity and a water contact angle (WCA) exceeding 90° requires a precise combination of surface roughness and low surface energy. The primary source of inspiration for creating hydrophobic surfaces has been lotus leaves, known as the “lotus effect”, attributed to their hierarchical micro- and nanoscale roughness combined with a waxy surface coating on the leaf surface [8]. Various techniques and specific chemicals have been investigated to impart durable water-repellent (DWR) properties to treated textiles [10]. For decades, a substantial portion of DWR chemistry has relied on polymeric per- and poly-fluoroalkyl substances (PFASs) due to their washing fastness and resistance to water and oil. In response to concerns regarding the adverse effects of long-chain PFASs, with a chain composed of eight or more carbon atoms, on human health and the environment, efforts have been made to substitute them with short-chain PFASs (with four to seven carbon atoms) or non-fluorinated alternatives [11,12]. However, emerging evidence indicates that short-chain PFASs also pose risks due to their persistence and toxicity, undergoing bioaccumulation and biomagnification within the food chain. Consequently, global policy is converging on a rapid phase-out of PFAS-based durable water-repellent (DWR) finishes for textiles. In the European Union, the universal restriction proposal submitted to the European Chemicals Agency in February 2023 would prohibit PFASs in apparel and other textile articles 18 months after adoption, with only narrow, time-limited derogations and an expected entry into force in 2026/27 [13]. Across the Atlantic, California’s Safer Clothes and Textiles Act (AB 1817) banned the manufacture and sale of PFAS-treated textiles from 1 January 2025, tightening the allowable total organic fluorine threshold to 50 ppm by 2027. France has gone further, outlawing PFASs in consumer clothing and footwear from 2026 and extending the ban to all textiles by 2030. These converging measures will eliminate the long-chain fluorocarbon chemistries that currently underpin most commercial DWR formulations, creating an urgent need for fluorine-free alternatives capable of meeting the impending regulatory limits without compromising performance [2,14]. This study addresses that need by evaluating a water-borne PDMS–silica hybrid finish applied to cotton and polyester, benchmarked against a state-of-the-art C6-fluoropolymer control [15].

The drive to replace fluorinated chemistries with environmentally benign alternatives has led researchers to explore various strategies, such as spray coating [16], radiation-induced graft polymerisation [17], chemical vapour deposition [18], layer-by-layer assembly [19], and plasma treatment [20]. Nevertheless, most methods mentioned above require significant investments, multiple and complex preparation steps, and long reaction times, which are severe disadvantages for practical applications.

The pad-dry-cure method has proven to be the most effective process for a potential industrial scale-up of fluorine-free waterproofing agents onto textiles, including long-chain alkanes, silicones, acrylates, sol–gel precursors, and modified polyurethanes [21,22]. Both long-chain alkanes and acrylate-based agents exhibit effective hydrophobic properties. However, their molecular structures, which contain numerous rigid segments, limit their flexibility and result in a worse hand feel of the fabric after treatment [23]. Due to their environmentally friendly nature and unique chemical composition, silicone-based water repellents are gaining increased attention. These molecules, characterised by long silicon–oxygen bonds, allow for significant rotational mobility. The strategic placement of methyl groups within the molecular structure reduces surface energy, thereby enhancing the water resistance of coated samples [24]. Furthermore, these methyl groups effectively mask the polarity of the silicon–oxygen backbone, reducing intermolecular attraction and providing a smooth texture upon application [25]. Nonetheless, this advantage is counterbalanced by the poor adhesion between silicone-based agents and treated fabrics, which reduces the durability of the finish after washing [26]. Unfortunately, they often fall short in abrasion resistance and durability compared to fluorinated counterparts. Recent advancements include the use of bio-based compounds, which demonstrate varying degrees of water repellence and durability [27]. Similar to silicone-based finishes, wax-based coatings derived from natural sources offer promising hydrophobicity but are prone to wear and limited thermal stability.

In recent years, nanotechnology and polymer science advancements have opened new avenues for developing innovative, environmentally sustainable water-repellent treatments [28]. Owing to its simplicity, adaptability, versatility and sustainability, sol–gel chemistry is extensively employed in fabricating functional and protective coatings across various industrial and application sectors, including textiles. This method facilitates the creation of surfaces with enhanced mechanical strength, chemical stability, and thermal resistance. It is a versatile synthetic pathway involving a two-step reaction (hydrolysis and condensation), typically initiated from (semi)metal alkoxides (e.g., tetraethoxysilane, tetramethoxysilane, and titanium tetraisopropoxide), resulting in the formation of fully inorganic or hybrid organic–inorganic coatings at or near ambient temperature [29]. In recent decades, the deposition of these coatings using alkoxysilane precursors on the surface of textile materials allows the development of a physical barrier acting as an insulator, thus improving the ordinary performances of the treated materials, such as flame retardancy [30], antimicrobial or UV radiation protection [31,32], dye fastness [33], anti-wrinkle finishing [34], hydrophobicity [21,35,36,37,38,39,40,41], and bio-molecule immobilisation [42]. To achieve sol–gel-based coatings with specific chemical–physical properties, the impact of the synthesis parameters on the final structure is crucial [43]. The sol composition is paramount as it governs the hydrolysis and condensation reactions, thereby ultimately determining the structural properties of the resultant material. The properties of the coatings are influenced not only by the nature of the chemical precursors, but also by the types of catalysts and solvents used, the reaction temperature, the ageing time of the sols, the stabilisation temperature, and the number of layers deposited [43,44,45]. As reported in the literature [46,47], the solvent phase acts as an intermediary between siloxanes and water, facilitating the uniform distribution of reactants during the hydrolysis and condensation reactions of sol–gel precursors and accelerating the rate of water evaporation. Employing a solvent with a high dielectric constant limits intermolecular interactions, consequently reducing the kinetics of condensation and diminishing the conversion rate of reactants to the gel form. Furthermore, the solvent affects the microstructure, film crystallisation, crystallite size, and properties, decelerating the hydrolysis rate and condensation kinetics [45,48]. Other studies indicate that increasing the reaction medium’s polarity modulates cation distribution within the crystalline network, thereby promoting the growth phase [46,48].

Currently, a significant obstacle to the extensive commercial application of silane chemistry is the poor durability of the resulting hydrophobic surfaces, particularly in terms of washing fastness. The durability of water-repellent coatings after washing, especially those applied to cotton, remains an issue. Laundering can reduce the hydrophobicity of treated fabric surfaces by disrupting the bonds formed by silanes and introducing contaminants such as residual surfactants and moisture [49]. Chemical softeners are surfactants with both hydrophilic and hydrophobic segments in their molecular structure. The hydrophobic segments may consist of long alkyl chains containing 16–22 carbon atoms, silicones, or polyethylene softeners. Textile softeners, including silicones, can be categorized based on their ionic nature into anionic, cationic, amphoteric, and non-ionic types [50]. Textiles treated with silicone softeners exhibit high softness, smoothness, a greasy feel, enhanced crease and abrasion resistance, improved tear strength, good sewability, excellent antistatic properties, good thermal stability, and a range of hydrophobic to hydrophilic properties [51]. In siloxanes, the alternating silicon and oxygen bonds establish a robust silicon–oxygen backbone, with organic groups attached to the silicon atoms. In commercial silicones, methyl groups are commonly present as organic substituents, thus resulting in polydimethylsiloxanes (PDMS). Due to their hybrid inorganic–organic chemical structure and the flexibility of siloxane bonds, silicones possess unique properties such as excellent dielectric characteristics, low-temperature flowability, low viscosity change with temperature, high compressibility, and spreadability, as well as thermal and oxidative stability [26]. Silicones exhibit a low glass transition temperature, elasticity, low surface tension, and film-forming capability. Their chain flexibility arises from the rotational freedom of the Si–O–Si linkages and the low interaction energies between the methyl groups [52]. As an example, aminopropyl-terminated polydimethylsiloxane (APT-PDMS) has been used as a chemical to prepare water-borne solutions for textile finishing, providing a “super soft” handle [53]. Strong hydrogen bonds between the hydroxyl or amino groups of fibres (in cellulose, wool, silk, and polyamide fibres) and the amino groups of modified silicone act as an anchor for the silicone, forming a uniformly distributed film on the fibre surface.

This research investigated the fabrication of a water-borne hydrophobic finish through a one-step sol–gel process conducted under acidic conditions. This finish was then applied to cotton and polyester textiles using the pad-dry-cure method. To evaluate the obtained performance, the proposed formulation was compared with a commercial fluorine-containing finishing, which was considered standard. Pristine and functionalized fabrics were compared, and their surface morphology was observed by scanning electron microscopy (SEM), while the chemical composition was investigated by energy-dispersive X-ray spectroscopy (EDX), attenuated total reflection–Fourier-transform infrared spectroscopy (ATR-FTIR), and Raman spectroscopy. In addition, thermal stability assessments were performed. To evaluate the wettability of the finished samples, static water contact angle values (WCA), dynamic wettability through spray test performances, and resistance to conventional water-based liquids were analysed. Additionally, the durability of the coatings was further investigated through washing cycles, as well as CIE L*a*b* and CIE whiteness index measurements. A comprehensive evaluation of the textile properties of coated fabrics was carried out by performing bending stiffness and water-vapour transmission rate assessments to evaluate their ability to retain comfort and breathability compared to uncoated samples.

The results demonstrated that the treated fabrics exhibited significant water-repellent properties, which were maintained even after multiple washing cycles. The effectiveness of fluorine-free coatings is thoroughly discussed based on the experimental evidence. The simplicity of the procedure and the promising results obtained for both cotton and polyester fabrics suggest its potential for industrial applications.

## 2. Materials and Methods

### 2.1. Materials

Scoured and bleached, 100% cotton (CO) and 100% polyester (PES) plain-weave fabrics, both with a mass per unit area of 120 g·m^−2^, were supplied by Albini group (Albino, Italy) and Stamperia di Martinengo (Martinengo, Italy), respectively. The selected textile samples were washed in 2% non-ionic detergent at pH 7 and 40 °C for 20 min, then rinsed several times with de-ionised water, dried, and finally conditioned in a desiccator under standard atmospheric pressure at 65 ± 4% relative humidity and 20 °C for at least 24 h before all experiments, in accordance with ISO 139:2005 [54]. A minimum of three textile fabric samples were prepared for each tested finish.

Triethoxy(octyl)silane (OS) (>98%), acetic acid, ethanol, and hydrochloric acid (all reagent grades) were purchased from Merck (Milan, Italy). Perfloro FPU BS, a commercial water-repellent finishing agent consisting of an aqueous emulsion with approximately 20 wt% concentration of fluorinated polyurethane oligomers, and “Catalizzatore is green”, a crosslinking agent with isocyanate groups, were generously provided by F.T.R. S.r.l. (Albano Sant’Alessandro, Italy). Aminopropyl-terminated polydimethylsiloxane (APT-PDMS) was kindly supplied by Lautex S.r.l. (Bergamo, Italy), emulsified with water at a 20% (*w*/*w*) concentration. All chemicals and reagents were used directly without further purification.

### 2.2. Preparation of Finishes and Their Application

The silica-containing finish for the water-repellent treatment of textiles was prepared by slowly adding triethoxy(octyl)silane (7 g) to an aminopropyl-terminated polydimethylsiloxane water emulsion (30 g, 70% *w*/*w* water content) under continuous stirring at room temperature. Thus, dropwise hydrochloric acid (HCl, 0.1 M, 1 mL) was added to promote the hydrolysis–condensation reaction. Under these conditions, the molar ratio of OS/H_2_O/HCl was approximately 1:128:0.004. Due to the protonation of aminopropyl groups present in the system, the final pH of the reaction mixture was adjusted and stabilised around 3. Moreover, 3.7 g of a crosslinking agent (trade name “Catalizzatore is green”) was added to the resulting mixture, which was then vigorously stirred at room temperature for 4 h.

The fluorine-based solution was prepared by mixing 20 g of Perfloro FPU BS (20% *w*/*w*) with 2 g of the crosslinking agent (Catalizzatore is green) and 100 g of distilled water. The components were thoroughly mixed to ensure uniformity. Cotton and polyester samples (20 cm × 30 cm) were impregnated separately three times with the aforementioned silica-based and fluorine-based solutions using a two-roll laboratory padder (Werner Mathis, Zurich, Switzerland), set to a nip pressure of 2 kg·cm^−2^ to achieve a weight pick-up of around 60%. After drying at 135 °C for 2 min, the fabrics (coded as CO_Si, CO_F for cotton and PES_Si, PES_F for polyester) were cured in a gravity convection oven at 170 °C for 1 min. Before all experiments, the samples were conditioned under standard atmospheric pressure at 65 ± 4% relative humidity (RH) and 20 ± 2 °C for at least 24 h. The add-on values A (%) were calculated as reported in Equation (1):

(1) $$A\;\left(\%\right)=\frac{\left(W_{treated}-W_{untreated}\right)}{W_{untreated}}\times 100$$

where (*W_untreated_*) and (*W_treated_*) represent the dry mass of the specimen before and after finishing, curing, and conditioning, respectively.

### 2.3. Washing Procedure

The coated fabric samples underwent 1–5 washing cycles according to the ISO 105-C10:2006 [55] standard method. The samples were immersed in a soap solution with a concentration of 5 g·L^−1^, preheated to 40 °C, and maintained at a liquor ratio of 50:1 for 30 min in a Mathis Labomat (Werner Mathis, Zurich, Switzerland). No surfactants were included to prevent their potential adsorption onto the fabrics, which could complicate the interpretation of the FTIR spectra. After every laundering cycle, washed samples were dried at 105 ± 3 °C in a forced-air oven until constant mass was reached (≤0.1 mg change in two successive weighings, typically 90 min for the fabric used), cooled to room temperature in a desiccator over fresh silica gel, and then weighed to the nearest 0.1 mg. The weight loss of fabrics after the washing cycles (*WLW*, wt%) with respect to the unwashed samples was calculated according to Equation (2):

(2) $$WLW\%=\frac{W_{treated}-W_{n}}{W_{treated}}\times 100$$

where (*W_treated_*) and (*W_n_*) denote the dry mass of the treated fabrics before and after n ISO 105-C10 wash cycles, respectively.

For each property, five replicates were taken, and the results are reported as mean ± SD in the corresponding tables and figures.

### 2.4. Characterisation Techniques

The morphology of both treated and untreated textile surfaces, along with EDX mapping, was obtained using a scanning electron microscope (SEM, Phenom ProX G6 Desktop SEM, Thermo Fisher Scientific, Waltham, MA, USA). The microscope was equipped with an energy-dispersive X-ray spectrometer (EDS), which analysed the distribution of chemical elements on the surfaces of treated and untreated cotton and polyester fabrics, operating at a voltage of 10 kV. Each sample was placed on aluminium sample holders through a graphite adhesive.

Colour difference and CIE whiteness index values between treated and untreated textile samples, as well as before and after washing cycles, were measured using a Datacolor Spectro 700 spectrophotometer (Datacolor, Giussano, Italy) following ISO 105-J02:1997 [56] and ISO 105-J03:2009 [57]. All measurements were realised using a USAV 6.6 mm aperture under a D65 illumination with a 10° standard observer, and the fabrics were folded 4 times to ensure that the image capture by the instrument was from the textile. The specular component was included for the CIE L*a*b* colour analysis, while for the CIE whiteness index, it was excluded. An average of three measurements was recorded for each sample.

This spectrophotometer enables precise colour analysis by quantifying colour differences (ΔE*) based on the measured CIE L*a*b coordinates, where L*, a*, and b* define lightness–darkness, red/green, and yellow/blue values, respectively, between a sample and a reference standard, applying the formula in Equation (3):

(3) $${\Delta E}^{*}=\sqrt{{\left({\Delta L}^{*}\right)}^{2}+{\left({\Delta a}^{*}\right)}^{2}+{\left({\Delta b}^{*}\right)}^{2}}$$

where ΔL*, Δa*, and Δb* are the differences in lightness, a*, and b* values.

Furthermore, according to the Colour Measurement Committee (CMC) system [58], an ∆E* value < 1 is the threshold below which samples are considered to have the same colour as the reference. For the whiteness index (W*_CIE_*), its value is calculated by Equation (4), where *X* (related to red/green sensibility), *Y* (the luminance), and *Z* (the blue sensibility) are the tristimulus values and *x_n_* and *y_n_* are the chromaticity coordinates for the diffuser under D65.

(4) $$W_{CIE}=Y+800\times \left(x_{n}-\frac{X}{X+Y+Z}\right)+1700\times \left(y_{n}-\frac{Y}{X+Y+Z}\right)$$

To compare the chemical surface modifications introduced by the coatings, FTIR spectra of both treated and untreated samples were acquired using a Thermo Avatar 370 spectrometer (Thermo Nicolet Corp., Madison, WI, USA), equipped with an attenuated total reflection diamond crystal accessory (ATR). Three replicate spectra were recorded for each sample in absorbance mode, between 4000 and 700 cm^−1^, based on 32 scans averaged at a resolution of 4 cm^−1^. The average spectra of the textiles were normalised to the bands at 1315 cm^−1^ (CH_2_ wagging of cellulose) and 1505 cm^−1^ (aromatic ring stretching of polyester), which are in a region where finishing absorptions are absent. The spectra were analysed using Omnic v 7.3 and processed with Origin v 7.0.

Raman spectroscopy was performed over the spectral range of 150–3900 cm^−1^ by an XploRA Plus Raman spectrometer (HORIBA Scientific, Kyoto, Japan) integrated with an Olympus confocal microscope, using a 600 groove/mm diffraction grating. The excitation source was a 785 nm laser focused on the sample through a 100× objective lens (numerical aperture of 0.9), delivering a laser power of 100 mW at the sample surface. The acquisition time for each spectrum was set to 50 s, and two accumulations were taken. The most representative spectra were selected for analysis and presentation. Each sample underwent three measurement repetitions to ensure reproducibility. A baseline correction was achieved for each Raman spectrum, followed by a min–max normalisation.

TGA of the samples was performed with a TA Instrument Discovery TGA 550 (TA Instrument, New Castle, DE, USA). The temperature ramp was set from 25 to 800 °C at a heating rate of 10 °C/min, with a flow rate and a balance flow of 90 mL·min^−1^ and 10 mL·min^−1^, respectively. The analyses were conducted in an air atmosphere. The experimental error was 0.5% for weight and 1 °C for temperature.

### 2.5. Evaluation of Water-Repellent Properties

The water-repellent properties of the cotton and polyester samples were initially assessed through contact angle measurements using a CCD camera (Imaging Source model “DMK 41BU02.H”) combined with a Navitar^®^ 1-50486 optical lens (Navitar, Rochester, NY, USA), achieving a typical spatial resolution of 4 µm/pixel. All measurements were conducted at room temperature. Contact angle values were determined using the standard sessile drop method, in which a drop (10 μL) of bi-distilled water was carefully placed on the fabric surface with a micro-syringe. Drop images were post-processed by fitting a circle to the drop contour near the contact point to obtain the contact angle. The average of five different locations on each sample was calculated for contact angle values, and the results are reported as mean ± SD in the corresponding tables and figures. A high contact angle indicates the effectiveness of the surface in repelling liquids. Due to the irregular surface of textiles, which affects the accuracy of large contact angle values and causes the immediate absorption of liquid drops as a result of their porous structure at lower contact angles, the wettability of textile surfaces was also evaluated using the American Association of Textile Chemists and Colorists AATCC 22-2017 Spray Test, providing a qualitative assessment of the fabrics’ ability to repel liquids of varying surface tensions.

According to the AATCC Test Method 22-2017 [59] (spray test, technically equivalent to ISO 4920 [60]), textile samples (200.0 mm × 200.0 mm) were cut from the test fabric and conditioned at 65 ± 2% relative humidity and 21 ± 1 °C for a minimum of 4 h before testing. The test sample was firmly secured in a 152.4 mm diameter hoop and then sprayed with 250 mL of distilled water at 20 ± 2 °C from the tester’s funnel for 25 to 30 s. After spraying, excess water was removed by tapping the specimen against a solid object. The water repellence of the fabric was assessed using a spray rating scale from 100 (ISO-5) to 0, where a rating of 100 indicates no adhesion of water droplets to the surface, while a rating of 0 signifies complete wetting of both the upper and lower surfaces of the tested fabric.

Finally, the water/alcohol drop test was used to assess the water repellence of the samples according to AATCC Test Method 193-2007 [61] (graded on a scale of 0–8, with grades based on the amount of isopropanol, increasing from 2% (grade 1) to 60% (grade 8)). Each fabric specimen was placed on a flat, horizontal surface, starting the test with the lowest-numbered standard set of eight water/alcohol solutions of varying surface tensions [62]. Three small droplets (with a diameter of approximately 5 mm) of the test liquid were gently applied to the fabric surface using a pipette. The water repellence was rated based on the highest-numbered test liquid, from 1 to 8, that did not spread on the fabric surface within 10 s.

### 2.6. Evaluation of Textile Properties

The stiffness of the coated cotton and polyester fabrics was evaluated as a representative parameter of their textile properties, as a decrease in stiffness provides valuable data on enhanced fabric softness. This mechanical characteristic is primarily influenced by factors such as yarn count, weave structure, and fabric areal density. Textile stiffness was assessed by determining the bending length (LB) according to the procedure described in DIN 53362 [63]. In this method, a fabric strip measuring 10.0 cm × 2.5 cm was advanced, along with a measuring bar, over the edge of a fixed horizontal bar. When the fabric deflected under its own weight beyond an angle of 41.5°, the corresponding overhang length was recorded, and the bending stiffness (B), expressed in cN·cm^2^, was subsequently calculated using the following Equation (5):

(5) $$B=0.00981\times m\times L^{3}$$

where *m* is the fabric mass per unit area (g·cm^−2^) and *L* is the measured overhang length (cm).

Each sample was measured in two directions and the mean values were calculated from five repetitions.

The water-vapour transmission rate (*WVTR*) test was carried out on finished fabrics to evaluate their ability to retain breathability despite the presence of coatings, which may hinder moisture transfer and thereby affect wearer comfort. The test was conducted in accordance with ASTM E96/E96M-22 [64], using the desiccant method. Circular fabric specimens, approximately 34 mm in diameter, were cut to fit into the opening of Becker-type test cups. Each cup was filled with 60 mL of silica gel (previously dried at 105 °C, cooled in a desiccator, and sieved) and weighed along with the fabric specimen before testing. The assembled cups were placed in the Water Vapour Lab equipment model 3395 (Mesdan SpA, Raffa, Italy) within a climate-controlled environment maintained at 23 °C ± 1 °C and 50% ± 1% relative humidity. The test duration was 16 h. The mass of each cup was recorded at the beginning of the test, after 7 h, and at the end of the exposure period. The *WVTR* was calculated according to Equation (6), as follows:

(6) $$WVTR=\frac{∆m}{A\times t}$$

where Δ*m* is the weight gain (g) due to water-vapour absorption by the desiccant, *A* is the exposed surface area of the fabric (m^2^), and *t* is the time (days) over which the weight gain was measured. The final results are expressed in g (m^2^·d)^−1^. All measurements were performed in triplicate to ensure reproducibility.

### 2.7. Statistical Analysis

Statistical analysis for contact angle measures was carried out by GraphPad Prism version 6 (GraphPad Software, La Jolla, CA, USA). Comparisons among groups were performed by the one-way ANOVA test, followed by Tukey’s post hoc test to compare the resulting data. Significance was retained when *p* < 0.05. Data are expressed as mean ± SD.

## 3. Results

### 3.1. Mechanism of the Polymeric Network Formation

For the development of eco-friendly hydrophobic formulations for both cotton and polyester fabrics, two sol–gel precursors were selected for their film-forming and hydrophobic properties as alternatives to perfluoroalkyl compounds. Triethoxy(octyl)silane (OS) features hydrophobic octyl groups covalently bonded to the siloxane backbone, thus acting as a hydrophobising agent. Aminopropyl-terminated polydimethylsiloxane (APT-PDMS), on the other hand, is a flexible, high-molecular-weight silicone polymer with terminal primary amine groups, offering excellent film-forming ability and elasticity, as well as inherent hydrophobicity. Triethoxy(octyl)silane undergoes rapid aqueous hydrolysis (t½ ≈ 2 h at 25 °C), yielding non-persistent octylsilanetriol and ethanol. At its solubility limit, it does not pose acute hazards to aquatic organisms (96 h LC_50_ > 0.055 mg/L for Oncorhynchus mykiss; 48 h EC_50_ > 0.049 mg/L for Daphnia magna), although it is not readily biodegradable and may form siloxane condensation products in the environment. According to ECHA [65], it is classified as Eye Irrit. 2 and Skin Irrit. 2, but is not considered PBT or vPvB. For aminopropyl-terminated PDMS, there are no harmonised classifications on the ECHA site [66], but industry self-classifications notify the substance as Eye Irrit. 2 and Skin Irrit. 2, as well as Aquatic Acute 1 and Aquatic Chronic 1. To enhance the overall environmental compatibility of the formulation, an isocyanate-based crosslinker was selected that complies with major textile eco-certifications, as it is included in the approved chemical lists of standards such as the Global Organic Textile Standard (GOTS, version 7.0:2023), Zero Discharge of Hazardous Chemicals (ZDHC, level 3), and bluesign^®^ [67].

Together, these organosilicon precursors represent a significantly lower environmental and human health risk than traditional fluoropolymers used in durable water-repellent (DWR) coatings, which are persistent, bioaccumulative, and associated with long-term ecological harm.

**Scheme 1:**
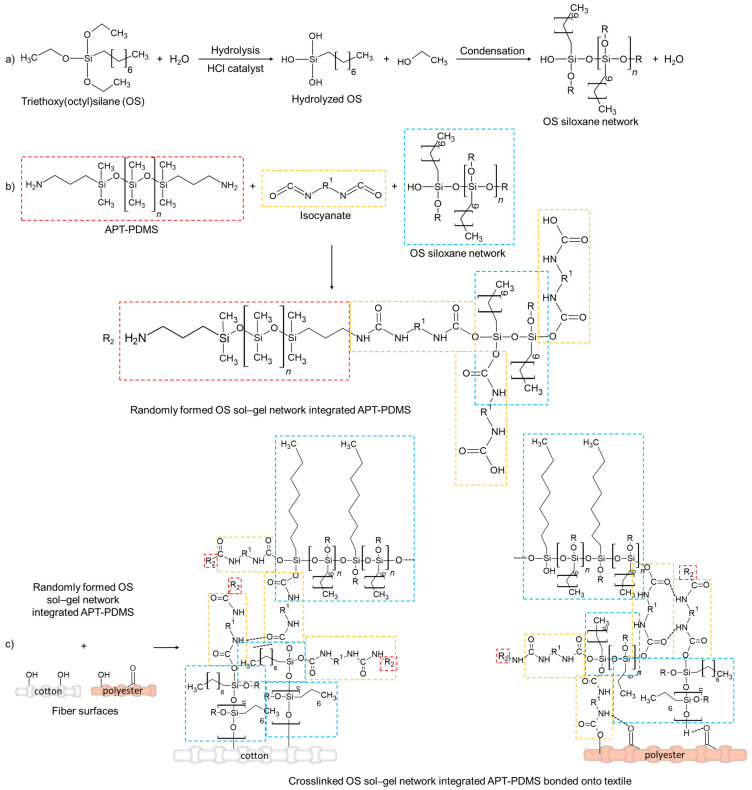
Proposed hydrolysis–condensation mechanism for the OS-based sol–gel network integrating APT-PDMS: (**a**) hydrolysis and condensation of the alkoxy groups of triethoxy(octyl)silane (OS) leading to the formation of Si–O–Si crosslinked structures; (**b**) subsequent isocyanate-assisted crosslinking between APT-PDMS and the OS sol–gel network, resulting in a randomly formed hybrid network containing both Si–O–Si and Si–O–C linkages, followed by (**c**) its application onto cotton and polyester fabrics. The blue, yellow and red dashed frames indicate OS siloxane network, isocyanate and APT-PDMS moieties, respectively.

According to the proposed reaction mechanism, triethoxy(octyl)silane undergoes acid-catalysed hydrolysis in the presence of water, in which the ethoxy groups (–OC_2_H_5_) are progressively replaced by hydroxyl groups, yielding silanol (Si–OH) species. Hydrochloric acid (HCl) is used to catalyse both the hydrolysis and the subsequent condensation reactions, facilitating the formation of a siloxane (Si–O–Si) crosslinked network (Scheme 1a). Simultaneously, APT-PDMS and an isocyanate-based crosslinker (trade name “Catalizzatore is green”) are introduced into the OS sol–gel solution (Scheme 1b). The terminal primary amine groups of APT-PDMS readily react with isocyanate moieties, forming stable urethane bonds. The isocyanate crosslinker may also react with hydroxyl groups in partially hydrolysed silanes or uncondensed silanols from the OS precursor, chemically integrating the flexible PDMS chains into the evolving siloxane matrix and contributing to the further crosslinking and structural reinforcement of the hybrid network. In this step, multiple reaction pathways are possible, resulting in heterogeneous crosslinked networks of Si–O–Si, Si–O–C, and urethane linkages, depending on local concentrations and functional group availability, which are randomly distributed.

The resulting sol–gel system comprises a hybrid organic–inorganic network that combines hydrophobic alkyl chains, flexible polysiloxane segments, and covalently crosslinked siloxane domains (Scheme 1b). This formulation is subsequently applied to cotton and polyester textile substrates. Durable adhesion and uniform coating distribution are achieved through covalent interactions between residual silanol or isocyanate groups and abundant surface hydroxyl groups on the cellulose fibres, forming stable Si–O–C bonds (Scheme 1c). For polyester substrates, which present fewer surface hydroxyl groups and a less reactive surface, adhesion mechanisms are more limited but still effective. In this case, adhesion may arise from covalent bonding with terminal hydroxyl or carboxyl groups, as well as from polar interactions and hydrogen bonding between the coating and the polymer surface (Scheme 1c).

**Scheme 2:**
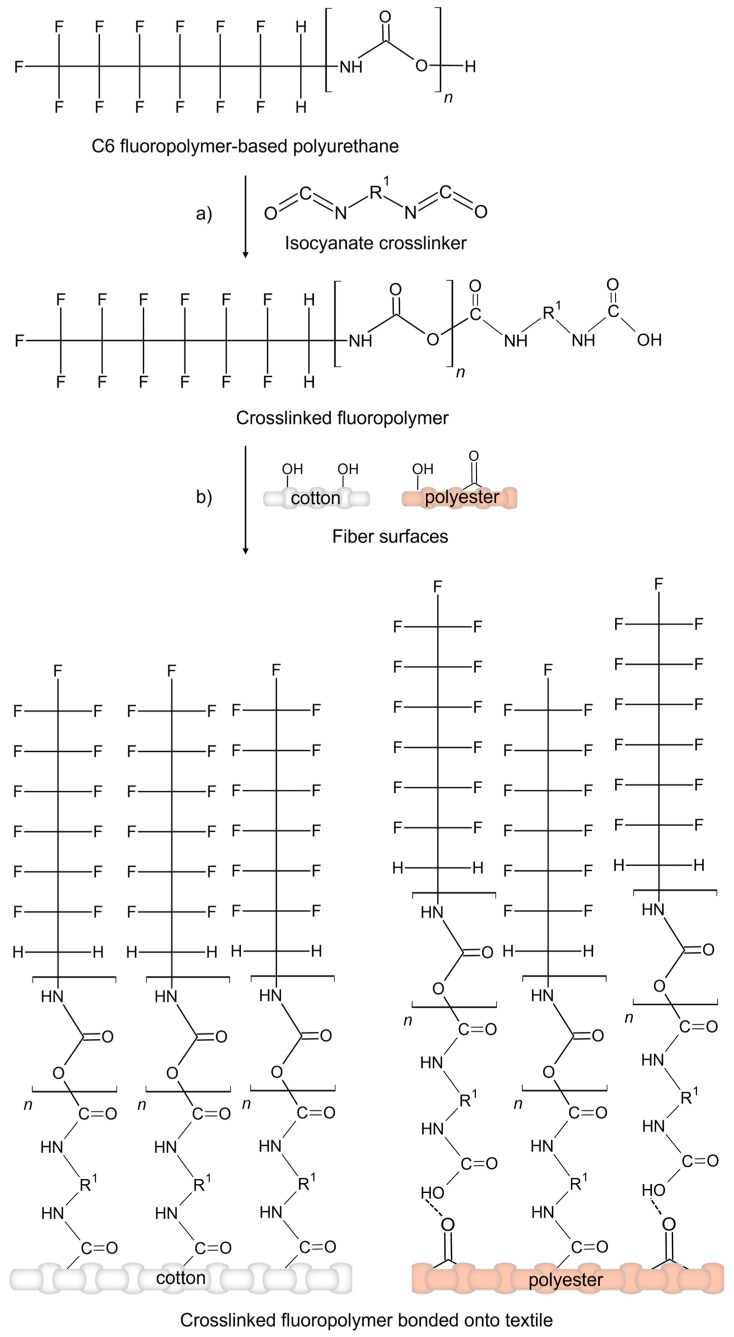
Proposed isocyanate-assisted crosslinking of C6 fluoropolymer-based polyurethane (**a**) and subsequent treatment of cotton and polyester fabrics (**b**).

On the other side, the C6 fluoropolymer-based polyurethane was combined with the isocyanate-based crosslinker. The isocyanate groups (-NCO) in the crosslinker readily undergo nucleophilic addition with the hydroxyl groups (-OH) present in the polyurethane backbone, leading to the formation of urethane bonds and the development of an extensively crosslinked polymer network. Moreover, the reaction leaves residual isocyanate functionalities available for subsequent interactions with substrate surfaces (Scheme 2a).

In the case of cellulose fibres, the high density of surface hydroxyl groups promotes covalent bonding with the coating through the formation of stable urethane (–NH–COO–) linkages, resulting in robust and durable adhesion (Scheme 2b). For polyester substrates, covalent bonding may occur via the reaction of isocyanate groups with terminal hydroxyl or carboxyl functionalities present on the polyester chains. Additionally, coating adhesion is further supported by polar interactions between the polyurethane matrix and ester carbonyls (Scheme 2b).

### 3.2. Spectroscopic Characterisation of Treated and Untreated Textiles

Attenuated total reflection–Fourier-transform infrared (ATR-FTIR) analyses were conducted between 4000 and 400 cm^−1^ to assess the structure and chemical composition of both the cotton and polyester samples, as well as the modifications introduced by the chemical finishes. Due to the low concentration of substances applied during the textile finishing, the infrared absorption characteristics of the chemicals applied to the fabrics overlap with the strong vibrational peaks of the cellulose and polyester macromolecules, making it difficult to recognise the differences between the spectra of the treated samples and pristine textiles.

**Figure 1:**
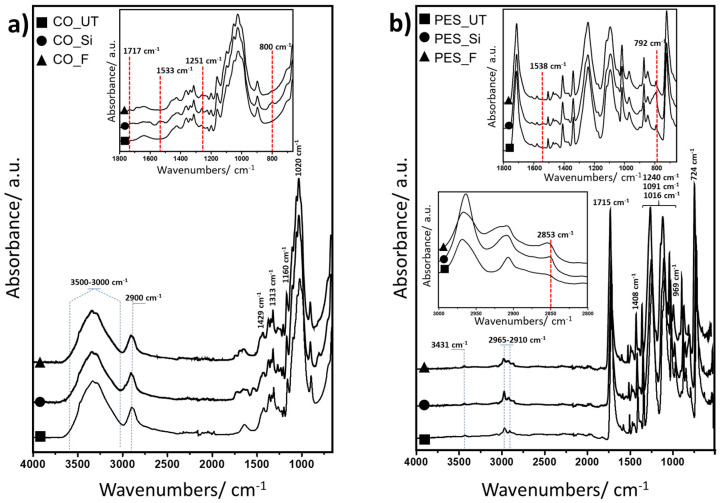
Normalised ATR FT-IR spectra of (**a**) cotton fabrics treated with silica-based (CO_Si) and fluorine-based (CO_F) chemicals, compared to untreated samples (CO_UT); (**b**) polyester fabrics treated with silica-based (PES_Si) and fluorine-based chemicals (PES_F), compared to untreated samples (PES_UT).

In the spectra of neat cotton (Figure 1a), the broad band around 3500–3000 cm^−1^ (O-H stretching) and the absorption bands at approximately 2900 cm^−1^ (C–H stretching) and 1429 cm^−1^ (C–H stretching) were consistent with the cellulose backbone [68]. Other significant peaks observed in the range of 1313, 1160, and 1020 cm^−1^ were attributed to bending and stretching vibrations of C–H, C–O, and COO present in the cellulose.

The deposition of silica-based coatings onto treated samples (Figure 1a) promotes a slight overall decrease in intensity at 3500–3000 cm^−1^, indicative of O–H stretching vibrations, suggesting the presence of a thin film on the fabric surface. Additionally, the Si–O–Si stretching band in the region 1150–1000 cm^−1^ is not discernible because of the overlap with characteristic absorption bands of cellulose. Nonetheless, the Si-O-Si bending mode at around 800 cm^−1^ confirmed the existence of a silane network in the treated cotton samples [69,70,71]. New absorption bands in the range of 1700–1500 cm^−1^, attributed to the formation of urethane linkages between isocyanate groups and cellulose hydroxyl groups, were observed at approximately 1717 cm^−1^ (C=O stretching) and 1533 cm^−1^ (N–H bending, amide II) [72], confirming the successful bonding of the coatings to the treated cellulose-based fabrics. On the other hand, the broad band around 3470 cm^−1^, ascribed to N–H stretching, overlaps with the O–H stretching of cellulose, making it difficult to resolve. Nonetheless, further evidence of the reaction between isocyanate groups and cellulose is provided by the absence of the intense absorption band at ~2257 cm^−1^, characteristic of free isocyanate groups, indicating their complete consumption during urethane formation. Subsequently, analysing the spectra of the fluorine-based coatings applied on cotton compared to the neat sample (Figure 1a), new bands at 1251 and 800 cm^−1^ are assigned to the CF_2_ symmetric stretching and bending. Weaker shoulders associated with the CF_3_ symmetric and degenerate asymmetric stretching modes can be observed at 1135 and 1236 cm^−1^, respectively [73]. These characteristic absorption peaks prove that fluoropolymer coatings have successful applications in cotton fabrics.

The FTIR spectrum of pristine polyester fabric is shown in Figure 1b. The peak at 3100 cm^−1^ belonged to a C-H stretching vibration, while the peaks at 2917 cm^−1^ and 2850 cm^−1^ were attributed to the characteristic vibrations of the -CH_3_ and -CH_2_ groups, respectively [74]. Other notable peaks include 1715 cm^−1^ (C=O stretching vibrations) and 1408 cm^−1^ (benzene ring stretching), as well as 1097 and 1040 cm^−1^ (C–O–C stretching vibrations) [75]. Additionally, the peaks at 1240 and 1016 cm^−1^ were associated with C–O stretching vibrations in ester and carboxylic acid groups. Furthermore, the peaks at 724 and 968 cm^−1^ indicated C–H bending vibrations, specifically out-of-plane and in-plane CH=CH bending, respectively. The Si–O–Si bending mode at around 800 cm^−1^ confirmed the existence of a silane network in the treated polyester samples, as shown in Figure 1b. In contrast to the treated cotton samples, the absorption bands typically associated with urethane bond formation are not discernible in the polyester substrates due to the high intensity and overlap of the characteristic polyester bands. Nevertheless, the absence of the absorption peak around 2257 cm^−1^, corresponding to unreacted isocyanate groups, confirms the occurrence of urethane linkage formation on the polyester surface. Additionally, the ATR FTIR spectra of fluorine-containing coatings on polyester samples, shown in Figure 1b, revealed new absorption bands at around 2800 and 1450 cm^−1^, assigned to characteristic vibrations of -CH_2_ and C–F groups, respectively [76], confirming the successful deposition of coatings.

The FTIR spectra of the cotton fabrics treated with the sol–gel-based coating after one, three, and five washing cycles did not exhibit significant changes compared to the unwashed sample, confirming the washing durability of the coating (Figure S1a). In contrast, fabrics treated with the fluorine-based coating showed a slight decrease in the intensity of the peaks associated with CF_2_ symmetric stretching and bending vibrations after the first wash, likely due to the removal of unreacted or weakly bound molecules (Figure S1b). The FTIR spectra of the polyester fabrics treated with the sol–gel-based coating (Figure S2a) and fluorine-based coating (Figure S2b) did not exhibit significant changes after one, three, and five washing cycles compared to the unwashed sample, confirming the washing durability of the coating.

**Figure 2:**
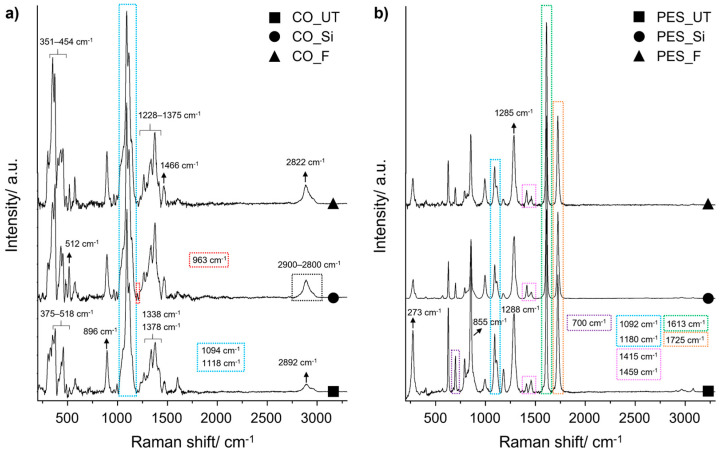
Raman spectra of cotton (**a**) and polyester (**b**) fabrics, both untreated and treated with silica-based and fluorine-based finishes, in the spectral range of 350–3500 cm^−1^.

As reported in the literature [77], the Raman spectrum of pristine cotton fabric (Figure 2a) displays several characteristic vibrational bands. Specifically, peaks at 375 cm^−1^, 436 cm^−1^, and 457 cm^−1^ are attributed to C–C–C ring deformations, while the band at around 900 cm^−1^ corresponds to CH skeletal rotations. Additional features include a band at 518 cm^−1^ associated with C–O–C glycosidic linkages, as well as symmetric and asymmetric stretching vibrations of C–O–C bonds at 1094 cm^−1^ and 1118 cm^−1^, respectively. CH_2_ vibrational modes are observed at 1338 and 1378 cm^−1^, while a C–H stretching band appears around 2900 cm^−1^.

According to previous studies [78], the spectrum does not show any detectable intensity corresponding to the O–H stretching vibrations of cellulose chains. These studies have demonstrated that O–H bonds are weakly polarizable and, therefore, typically undetectable by Raman spectroscopy.

The distinctive absorption bands mentioned above are also present in the Raman spectrum of cotton modified with silica-based and fluorine-containing coatings. The Raman spectra of the hydrophobic silica coating (Figure 2a, CO_Si) exhibit characteristic bands associated with silanol (Si–OH) groups. A broad band observed in the 2900–3800 cm^−1^ region corresponds to Si–OH stretching vibrations, while a weak band appearing at 963 cm^−1^ is attributed to the Si-O stretching vibration. In addition, the band at 512 cm^−1^ is ascribed to Si-O-Si symmetric stretching [79]. Moreover, C–F vibrational bands were identified in the Raman spectra of cotton samples containing fluoroalkyl chains, although they overlapped with those of the cotton substrate (Figure 2a). Indeed, a strong increase in the intensity of Raman bands in the range of 350–485 cm^−1^ was observed and ascribed to the C–F stretching vibrations of the CF_3_ group. Additional C–F vibrational modes from the –CF_3_ and –CF_2_ groups appeared around 1113 cm^−1^, in the range of 1228–1375 cm^−1^, and at 1466 cm^−1^; these overlapped with bands corresponding to C–C or C–H vibrations but remained recognisable within the spectra [79,80,81,82]. The Raman spectrum of neat polyester (Figure 2b) is characterised by two prominent and intense bands at 1613 cm^−1^, assigned to C–C stretching within the aromatic ring, and 1725 cm^−1^, corresponding to carbonyl (C=O) stretching vibrations [83]. Additional characteristic vibrational bands include a skeletal C–C deformation at 273 cm^−1^, a ring C–C stretching mode at 700 cm^−1^, and bending vibrations involving C–C and C–O–C at 855 cm^−1^. Stretching vibrations of C–C are observed at 1092 cm^−1^, while a ring C–C stretch appears at 1180 cm^−1^. The band at 1288 cm^−1^ is assigned to C–O–O stretching, and CH_2_ bending modes are evident at around 1415 cm^−1^ and 1459 cm^−1^. No new absorption bands are evident in either silica- or fluorine-treated polyester fabrics compared to the untreated polyester; however, a general decrease in the intensity of the characteristic bands is observed following the deposition of both coatings.

### 3.3. Effect of Surface Treatments on the Morphology and Optical Properties of Textiles

**Figure 3:**
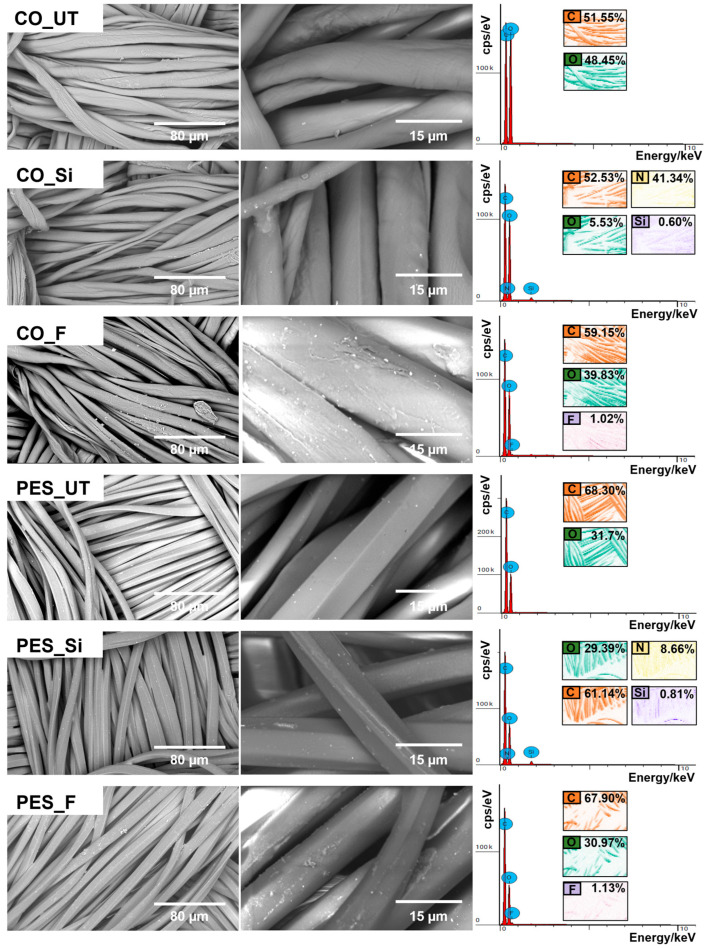
SEM images of untreated (CO_UT), silica-finished (CO_Si), and fluorine-finished (CO_F) cotton samples, and untreated (PES_UT), silica-finished (PES_Si), and fluorine-finished (PES_F) polyester samples at different magnifications and related EDS analysis.

The SEM-EDS investigation into untreated and treated fibres is reported in Figure 3. The pristine cotton fibres are relatively rough, exhibiting numerous natural cracks and slender grooves, along with typical convolutions resulting from the spiralling of cellulose fibrils. Although the ×10,000-scale micrographs (Figure 3) cannot resolve the nanometre-scale film thickness, the uniform change in surface contrast together with the nodular micro-texture suggests that the SiO_2_/PDMS finish forms a conformal layer on the fibre surface, whereas the fluoropolymer yields a smoother bead-like morphology. This interpretation is consistent with analogous coatings applied to cotton and polyester fabrics [84]. Recognition of the weave structure and individual yarns in the treated samples indicates that the applied finish did not produce a continuous, thick membrane on the fabric surface. Energy-dispersive spectroscopy (EDS), employed to quantitatively analyse the elemental distribution on the surface and examine the changes in elemental content before and after the coatings were applied, confirmed the presence of silicone and fluorine on both treated surfaces, in addition to carbon and oxygen, which are the only elements observed in the uncoated cellulose-based samples. The untreated PES fabric displays a characteristic slightly polygonal (four- to six-lobed) cross-section generated by melt-spinning through a slot-type spinneret, with smooth longitudinal surfaces devoid of particulate contaminants (Figure 3). In the PES_F sample, the fluoropolymer produces a continuous, crack-free film with a fine nodular micro-texture (Figure 3), fully covering the underlying fibre and thereby increasing surface roughness relative to the untreated control. Although the individual coating layers are below the SEM resolution at ×10,000, their presence is evident from the continuous change in surface contrast and the appearance of a fine nodular texture (Figure 3), indicating that the hybrid or fluoropolymer film uniformly blankets the fibres. Indeed, the corresponding EDS analysis reveals a higher silicone and fluorine content than that of the coated cotton fabric. Notably, for both coated polyester samples, the measured atomic mass concentrations of Si and F were 0.81 and 1.13, respectively, exceeding the values of 0.60 and 1.02 recorded in treated cotton fabrics. This result suggests stronger adhesion between the coating and the polyester fabric surface than with cotton, which could enhance the properties of the coatings applied to synthetic textiles.

In order to evaluate the influence of the applied coatings on the visual appearance of the textiles, optical properties were assessed through both CIE L*a*b* colourimetric and whiteness analysis. The colour differences (ΔE*) and whiteness index values (W*_CIE_*) between treated and untreated samples were calculated (Table 1), providing a quantitative measure of the coating-induced chromatic variations. The analysis of cotton (CO) and polyester (PES) fabrics demonstrated that surface treatments exert a notable influence on their optical properties, with the extent of variation depending on both the fibre composition and the type of functionalisation applied. In both fibres, cotton and PES, the application of a sol–gel coating or the fluorine-based treatment decreased their whiteness index. The untreated cotton had a whiteness colour index estimate (W*_CIE_*) of 125.40. When a silica-based sol–gel finish was applied, this value decreased to 108.85, representing an approximate 13% reduction. In contrast, the fluorinated finish resulted in a smaller decrease, lowering the W*_CIE_* to 117.48. For polyester, the initial W*_CIE_* was 76.94. The sol–gel coating reduced this value to 67.28, while the fluorinated coating brought it down to 71.67.

One reason that the silica-based sol–gel finishes had a higher W*_CIE_* reduction could be attributed to the PDMS structure in the sol–gel that changes the fabric’s surface, making it smoother and more transparent, and reducing the light scattering properties of the fabric [85,86]. Another factor could be ascribed to the combined influence of the aliphatic poly-isocyanate cross-linker and the mildly acidic bath: on both cotton and polyester, urethane linkages formed during curing introduce a faint yellow hue, while the low pH transiently protonates carbonyl groups and accelerates the minor hydrolysis of ester moieties, together diminishing diffuse reflectance.

On the other hand, colourimetric analysis showed that, in the case of cotton, the application of a sol–gel coating (CO_Si) induced a perceptible colour change (ΔE* = 2.29), primarily associated with a reduction in redness (a* = 2.64) and an increase in blueness (b* = −8.56).

Throughout routine laundering, the whiteness of the fabrics was gradually restored. For cotton, after one, three, and five wash cycles, the sol–gel samples increased to 114.93, 119.62, and approximately 119, respectively. Meanwhile, the fluorinated samples surpassed the initial untreated value, reaching 125.16, 126.95, and 129.35 after the same number of cycles. In the case of polyester, after five laundering cycles, the values for sol–gel finish and for the fluorinated finish were 66.35 and 75.31, respectively, where the latter almost recovered the untreated value. The reasons behind the recovery in the W*_CIE_* value could include the following: (i) The washing process can damage the surface of the fabrics, removing some of the deposited PDMS on it. This, in turn, increases the roughness of the fabrics (higher light scatter effect) and reduces the glossy effect of the PDMS. (ii) Neutral washing effectively removes unreacted oligomers and neutralises any residual acidity, allowing the whiteness of the sol–gel-finished fabrics to approach that of the uncoated textiles. In some cases, the fluorinated finish even surpasses the whiteness of the untreated fabrics. As a result, after three to five washing cycles, the difference between the two finishing systems remains minimal.

On the other hand, after laundering, ΔE* values showed only moderate fluctuations, indicating the durability of the hydrophobic finishes after repeated home-laundering cycles. These minor chromatic shifts suggest that the sol–gel layer remains visually stable during washing, although slight changes may arise due to the partial removal or redistribution of the coating on the fibre surface [87].

Cotton treated with the fluorinated coating (CO_F) exhibited minimal deviation from the untreated control (ΔE* = 0.50), reflecting a negligible impact on the fabric’s initial visual characteristics. Following laundering, ΔE* increased moderately after three washing cycles, then slightly decreased after five, confirming the coating’s strong visual stability. These findings align with literature reports indicating minimal pigment interference from hydrophobic fluorinated compounds [88].

For polyester samples, the sol–gel coating (PES_Si) led to a slight increase in the yellow component (b* = 3.02), accompanied by a subtle shift toward greener tones (a* = −0.85). The corresponding ΔE* value of 1.32 denotes a perceptible yet limited chromatic alteration. Notably, ΔE* values decreased consistently with additional washing cycles, suggesting a partial recovery of the fabric’s original colour characteristics. This trend is likely attributable to the gradual leaching or redistribution of the sol–gel network during laundering, which may expose more of the underlying fibre surface [89].

The fluorinated coating applied to polyester (PES_F) induced only minimal colour variations (ΔE* = 0.51), and subsequent washing further stabilised the colourimetric parameters. This behaviour suggests that the fluorinated finish is highly resistant to washing-induced degradation and does not significantly alter the visual appearance of polyester, corroborating previous studies on the durability of per-fluorinated treatments in textile applications [90]. Thus, the modest ΔE* values observed after laundering confirm that the coatings do not introduce perceptible colour defects, while the chemical and wetting analyses demonstrate their presence and durability.

The observed differences between cotton and polyester can be largely attributed to their distinct surface chemistries, which affect both the adhesion of the coating and the resulting optical response. Beyond chemical interactions, physical factors—such as surface roughness and curing conditions—also play a significant role in both colourimetric and whiteness index outcomes. Increased surface roughness can scatter incident light, subtly modifying the perceived hue. Moreover, the thermal curing process required for the development of the silica network may cause slight yellowing, particularly in cellulose-based fibres like cotton, due to thermal degradation. These factors collectively contribute to the moderate yet measurable chromatic shifts observed after both treatment and laundering. Nevertheless, the modest colour differences measured here (ΔE* < 1.32 for PES and ΔE* < 2.29 for cotton) confirm that the finishes exert only a limited overall impact on visual appearance; in cotton, the slight cream tint associated with ΔE* > 1 is perceptible to the naked eye but remains within the tolerance generally regarded as acceptable for white apparel fabrics.

### 3.4. Thermal Characterisation of Treated and Untreated Textiles

**Figure 4:**
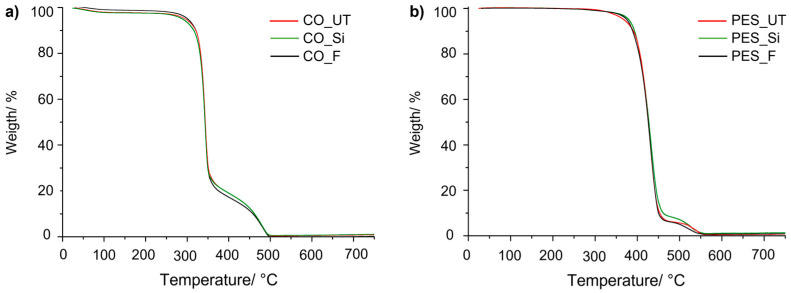
TGA curves of cotton (**a**) and polyester (**b**) fabrics, both untreated and treated with silica-based and fluorine-based finishes.

TGA was performed solely to confirm that the <≈2 wt% add-on of either finish leaves the pyrolytic stability of the base fabrics essentially unchanged. In agreement with the low loading, the derivative curves of treated and untreated samples superimpose within the instrumental uncertainty (onset T_5%_ and T_max_ shifts ≤ 4 °C), indicating that the coatings do not influence the thermal degradation pathway of either cotton or PES. The corresponding thermograms are presented in Figure 4a,b for cotton (CO) and polyester (PES), respectively. The TGA curve of untreated cotton fabric (CO_UT) exhibits a typical two-step degradation profile, consistent with the thermal behaviour of cellulose-based samples. The temperature at 10% weight loss (T_onset10%_) occurs at approximately 315 °C, whereas the maximum degradation rate (T_max1_) is noted around 365 °C, associated with the pyrolytic breakdown of the cellulose structure. At 800 °C, the final char residue is approximately 1.5%, reflecting the predominantly organic composition of the material.

In the case of polyester fabrics, the untreated sample (PES_UT) exhibits a single-step degradation process, with T_onset10%_ at approximately 395 °C and T_max1_ near 430 °C, indicating the breakdown of ester linkages and aromatic chains. The final residue at 800 °C is about 5%, as expected for this synthetic polymer.

The thermal behaviour of both cotton and polyester fabrics treated with silica- (CO_Si and PES_Si, respectively) and fluorine-based (CO_F and PES_F, respectively) finishes remains unchanged. The onset and peak degradation temperatures remain unaffected (only a marginal variation of around 6% was observed), suggesting that the thermal stability of the textile substrates is preserved post-treatment. The observed increases in char residue for the silica-treated samples are consistent with the incorporation of non-combustible inorganic components, while the slight increase in residue for the fluorinated samples likely reflects the limited presence of thermally resistant fluorinated compounds. These results confirm that the coatings, while effective in imparting water repellency, do not compromise the thermal behaviour of the textiles.

### 3.5. Assessment and Durability of Water-Repellent Properties

In this study, a novel water-repellent finish for cotton and polyester fabrics was designed and developed using a sol–gel technique without alcohol. In this method, the precursor was directly hydrolysed with emulsified amino-functionalized polydimethylsiloxane.

The adhesion of the coatings to fabric substrates, in comparison to untreated textiles, was evaluated by determining the add-on percentage (wt%), as defined in Equation (1). Similarly, the durability of the coatings was assessed based on weight loss (*WLW*, wt%), calculated using Equation (2). The results of these calculations are presented in Table 2.

Among the coatings, CO_Si exhibits the highest initial add-on value (1.78 wt%), indicating superior coating uptake compared to the other formulations. However, its durability appears moderate, with *WLW* increasing progressively after 5 washes. In contrast, CO_F shows the lowest add-on value (0.39 wt%), and correspondingly, a relatively stable and low *WLW* profile, suggesting limited coating uptake and minimal leaching. The PES_Si coating demonstrates a moderate add-on value (0.92 wt%) but shows a significant increase in *WLW* over successive washings (after five cycles). This trend indicates lower wash resistance, possibly due to weaker interactions with the fabric substrate. Meanwhile, PES_F displays a similar add-on value (0.96 wt%) but much better washing stability, with *WLW* values plateauing around 1.58% after five cycles. Overall, these results highlight a trade-off between coating uptake and durability, with formulations applied to F substrates generally exhibiting better resistance to washing, whereas Si-based substrates tend to retain higher initial coating loads but with more substantial losses over time.

**Figure 5:**
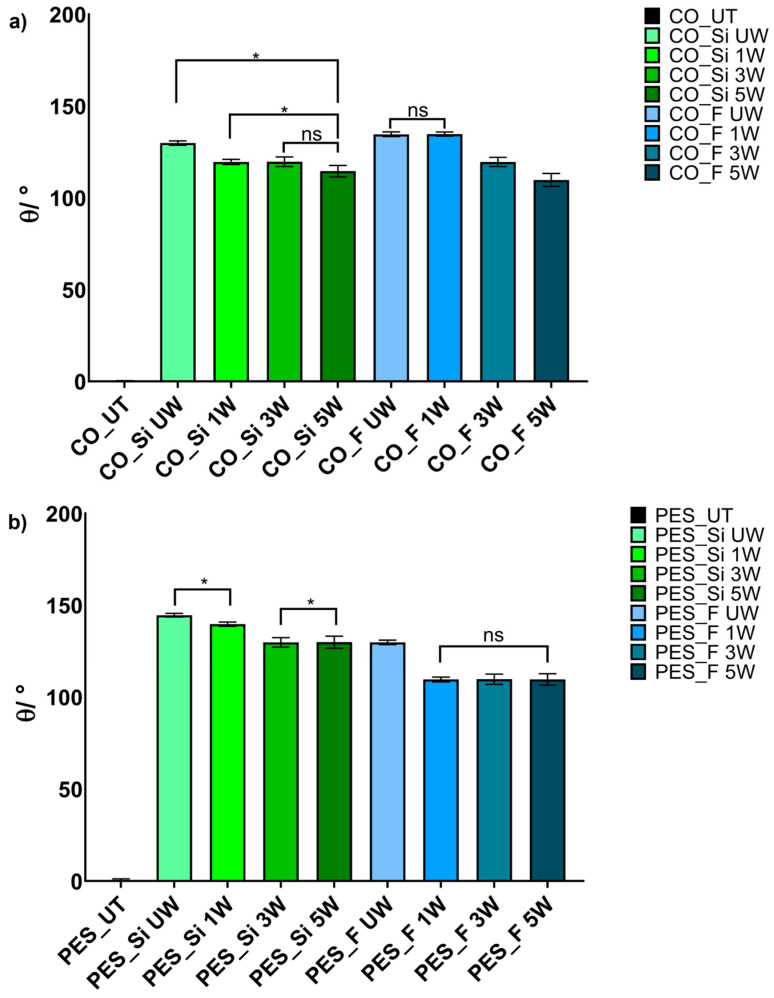
Contact angle (θ) of cotton (**a**) and polyester (**b**) before and after treatments and washing cycles. Samples coded as follows: UT: untreated; Si: SiO_2_/PDMS-finished; F: fluoropolymer-finished; 1W, 3W, 5W: after 1, 3, 5 ISO 105-C10 wash cycles. Data are expressed as means ± SD. (ns, *p* > 0.05; * *p* < 0.05).

To assess the performance of the developed treatment, the hydrophobic properties of the treated fabrics were compared with those of identical fabrics finished with the commercial fluorine-based product. The evaluation was conducted by measuring the static water contact angle (θ) using the sessile drop method, a standard technique for assessing surface wettability. The hydrophobic performance of the fabrics after each modification step is presented in Figure 5. The results demonstrated that initially super-hydrophilic cotton could be effectively rendered hydrophobic, with water contact angles exceeding 120° under all tested conditions (Figure 5a). Therefore, the fluorinated finish yielded the highest contact angle values for CO and for PES, indicating excellent static repellence. ANOVA analysis revealed a statistically significant difference (*p* < 0.05) between the cotton samples treated with silica-based and fluorine-based finishes. A reduction in mean contact angle values was generally observed after washing cycles. However, the decrease was gradual even after five washing cycles in the silica-finished cotton fabrics. This gradual reduction of 15° over five cycles suggests a moderate loss of surface hydrophobicity, which may be attributed to partial removal or surface restructuring of the silica-based coating. No significant decrease was observed after one washing cycle for the fluorine-treated samples. For the polyester fabrics (Figure 5b), both treatments successfully imparted hydrophobic properties, with water contact angles exceeding 130°. A statistically significant difference was observed between the silica- and fluorine-based treatments (*p* < 0.0001). The superior θ value observed on polyester for the Si-based treatment indicates a synergistic effect between the surface chemistry of PES and the silica-based coating, which may enhance surface roughness. After multiple washing cycles, silica-treated polyester showed only minor changes in contact angle, while the fluorine-treated samples maintained their hydrophobic performance with no statistically significant variation even after five washing cycles. These values indicate a strong retention of surface structuring and the hydrophobic function of the finish, particularly on the synthetic substrate.

In the Supplementary Materials, a semi-log representation is provided to visualise the quasi-exponential decay of surface hydrophobicity under home-laundering (Figure S3). Although the dataset comprises only three wash points beyond the origin, the linearity of the plots supports a first-order approximation and highlights the durability gap between the two chemistries: the SiO_2_/PDMS finish displays steeper slopes, whereas the fluoropolymer yields gentler slopes. Although the confidence intervals are wide because of limited n, these findings corroborate the qualitative conclusion drawn in the main text: the fluorinated film is roughly three times more wash-resistant than the sol–gel hybrid, while both retain the majority of their initial contact angle after five domestic washes. A full kinetic study with a denser laundering profile will be reported in future work.

Furthermore, the hydrophobic properties of treated fabrics were evaluated through the spray test, designed to assess surface wetting under dynamic conditions according to ISO 4920, as well as the water repellence test according to AATCC TEST METHOD 193-2007. These investigations were even performed on samples after one, three, and five washing cycles to examine the durability of each finish under realistic laundering conditions. All obtained results are listed in Table 3.

The spray test and water/alcohol repellence test confirmed that untreated cotton and polyester fabrics completely lacked water repellence, with both tests scoring 0, indicating full wetting. Although PET is intrinsically hydrophobic, the scoured plain-weave PES fabric used here is macroscopically hydrophilic: water is wicked into the yarn interstices instantaneously (static CA < 5°, ISO 4920 spray rating 0), in agreement with previous reports on similar untreated PES textiles [35]. These findings highlight the need for functional surface treatments in applications requiring moisture resistance. After treatment, both silica-based (_Si) and fluorinated (_F) finishes demonstrated excellent hydrophobicity, achieving the highest possible scores: 100 (ISO-5) in the spray test and 8 in the AATCC 193-2007 test. These values exceed the standard performance threshold (ISO-2 = 70) required for functional outerwear. Durability under laundering was assessed after one, three, and five wash cycles on both cotton and polyester, using contact angle, spray, and water/alcohol repellence tests (Table 3). On cotton, the _Si coating maintained a spray rating of 100 through three washes, decreasing slightly to 90 (ISO-4) after five, while the water/alcohol resistance reached the value of 7 after one wash. This suggests good durability, despite minor surface degradation. On polyester, the _Si finishing performed even better, maintaining a spray rating of 100 and only a minor drop in repellence (from 8 to 7) after three washes. In comparison, the _F-treated cotton showed reduced durability under dynamic conditions, with spray ratings dropping from 100 to 70 over five washes. Water/alcohol repellence decreased from 8 to 7, accompanied by a more noticeable decline in contact angle, indicating a more fragile hydrophobic layer. On polyester, the _F finish retained a spray rating of 100 and consistent water/alcohol repellence (score of 8), outperforming the _Si treatment in that test. This may be due to the inherently low surface energy of fluorinated groups, which remain effective even after mechanical stress and exposure to detergents.

**Figure 6:**
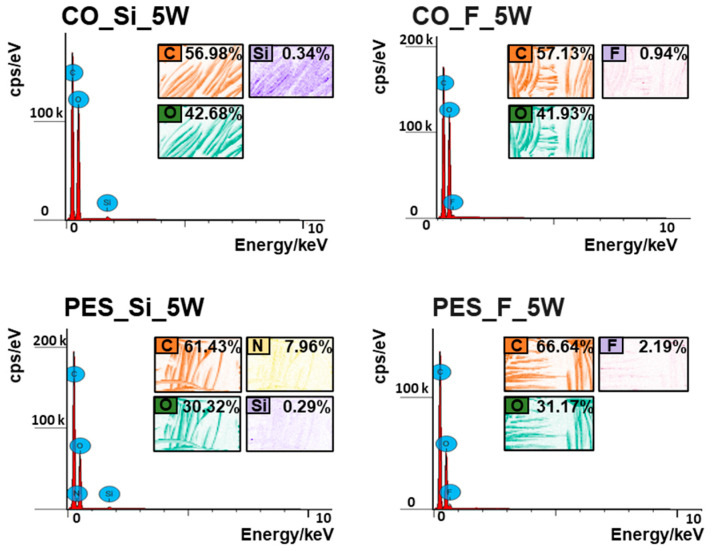
EDS elemental spectra and corresponding maps of silica-based and fluorine-based finishes on cotton and polyester fabrics after 5 ISO 105-C10 wash cycles.

To confirm the durable attachment of coatings to the fabric surface, EDS spectra were performed on samples after five washing cycles (Figure 6).

The EDS analysis (Figure 6) reveals the Si signal along the entire fibre length for both cotton and PES finished with the SiO_2_/PDMS sol–gel, even after five laundering cycles, whereas the untreated controls show no Si peak (Figure 3). Likewise, a uniform F-Kα map persists on fluoropolymer-finished fabrics in agreement with the performance observed by water contact angle, spray and water/alcohol repellence tests, thereby confirming that both finishes remain firmly anchored after repeated washing.

### 3.6. Textile Properties

The impact of the applied coatings on the physical properties of all tested fabrics was systematically evaluated. Chemical finishing treatments can often lead to undesirable alterations in fabric performance, mainly resulting in diminished tensile characteristics. The chemical agents employed in this study promoted the formation of chemical bonds with the polymer surface, which may have adversely affected the mechanical properties and comfort-related performance of the textile materials. Indeed, excessive stiffness resulting from the water-repellent treatment can adversely affect the flexibility and wearability characteristics of textile materials. Therefore, maintaining an appropriate balance between functional performance and mechanical softness is essential. Furthermore, comfort remains a key evaluation parameter, particularly in technical textile applications. Water-vapour permeability (WVP), in particular, is a critical factor, as it directly influences the breathability of fabrics treated with water-repellent finishes. Consequently, assessing the textile properties post-finishing is crucial to ensure that enhancements in specific attributes do not compromise others. The textile properties, including stiffness and water-vapour permeability, were examined, and the corresponding results are summarized in Table 4.

The untreated cotton and polyester fabrics exhibited bending stiffness values of 12.0 ± 0.5 cN·cm^2^ and 9.0 ± 0.4 cN·cm^2^, respectively. These values reflect the inherent structural characteristics of the fibres, with cotton displaying slightly higher stiffness due to its natural morphology and yarn configuration. Regarding the finishing applications, the results indicate a certain increase in bending stiffness in the treated cotton and polyester fabrics compared to their untreated counterparts (Table 4). The sol–gel treatment resulted in a slight increase, likely due to the formation of a thin silica-based layer on the fibre surface. The fluorocarbon treatment produced the highest stiffness values, suggesting the presence of a denser and more rigid polymeric coating. These remained within a range that did not drastically alter the mechanical flexibility of the fabrics, particularly in the case of the sol–gel-treated samples. These findings suggest that hydrophobic functionalisation can be achieved while minimally compromising the bending behaviour, especially when using sol–gel systems.

Water-vapour transmission rate (*WVTR*) measurements were performed on both untreated and treated cotton and polyester fabrics to evaluate their breathability. Assessing breathability, along with stiffness, is essential for determining the comfort properties of garments, which should be preserved even after applying functional finishing treatments. The results reported in Table 4 indicated that the uncoated samples exhibited *WVTR* values of 905 ± 31 g·(m^2^·d)^−1^ for cotton and 797 ± 28 g·(m^2^·d)^−1^ for polyester. After applying the silica-based coatings, the treated cotton and polyester fabrics showed *WVTR* values of 869 ± 15 g·(m^2^·d)^−1^ and 763 ± 17 g·(m^2^·d)^−1^, respectively, indicating nearly the same water-vapour permeability as pristine textiles. The fluorine-based coatings resulted in a more significant reduction in *WVTR*, thus leading to 810 g ± 21 g·(m^2^·d)^−1^ and 708 ± 18 g·(m^2^·d)^−1^ values for fluorine-treated cotton and polyester, respectively.

However, these findings suggest that the applied finishes did not compromise the breathability of the neat fabrics, as the coatings did not form a film on the textile surface. Instead, they promoted films on each individual fibre, preserving the comfort-related properties of the developed water-repellent treatment textiles.

In conclusion, the sol–gel treatment slightly enhances the bending stiffness of both cotton and polyester fabrics, while only modestly reducing the water-vapour transmission rate. This balance makes sol–gel functionalisation a promising approach for applications requiring mechanical performance and breathability.

### 3.7. Limitations and Research Outlook

Despite the promising results, the current findings are subject to certain limitations. While domestic laundering was used as the primary durability assessment in this study, it did not account for other critical real-world stressors such as UV exposure, mineral and tap water contact, and mechanical abrasion. These factors could significantly influence the long-term durability of water-repellent textile coatings. For example, prolonged UV radiation can photochemically degrade hydrophobic coating polymers, mineral deposits from non-purified water may create residues that diminish surface repellency, and repeated mechanical friction or abrasion can physically wear away the protective coating layer.

Acknowledging these limitations, future work should expand durability testing to include dedicated UV ageing experiments, mineral and tap water exposure, and standardised abrasion tests. Additionally, a full kinetic study with a denser laundering profile will be addressed.

Future work will also include nanoscale characterisation techniques. In particular, to directly visualise the coating nanostructure and corroborate its inferred continuity, field-emission SEM or AFM will be employed.

Moreover, a comprehensive life-cycle assessment, particularly regarding the fate of silicone residues at end-of-life, still needs to be conducted as part of future investigations.

This broader approach will help validate the coating’s long-term performance under field-relevant conditions.

## 4. Conclusions

This work demonstrates that a single-step, water-borne PDMS–silica sol–gel finish can deliver durable water repellence to bleached cotton and polyester fabrics without resorting to PFAS chemistry. The aqueous hydrolysis of triethoxy(octyl)silane in an amino-PDMS dispersion forms a conformal, chemically stable coating, as confirmed by FTIR, Raman spectroscopy, and EDS mapping. The treated textiles exhibit static contact angles of approximately 130° on cotton and 145° on polyester, while the CIE whiteness index remains within ten units of the untreated controls. This indicates that hydrophobicity is achieved without a noticeable loss of whiteness. After five ISO 105-C10 home-laundering cycles, the sol–gel layer retains about 80% of its initial contact angle. Crucially, the finish leaves water-vapour permeability practically unchanged (≤5% loss), adds less than 0.9 cN·cm^2^ to bending stiffness, and does not affect the substrates’ thermal stability at the ≤2 wt% add-on employed. Although long-term UV ageing, exposure to mineral water, and mechanical abrasion still need to be explored, the present results confirm that a fluorine-free, industrially scalable sol–gel route can satisfy forthcoming PFAS restrictions while preserving the breathability, handle, and optical appearance demanded by high-performance apparel and technical textiles.

## Figures and Tables

**Table 1:** CIE L*a*b* coordinates (mean ± SD, *n* = 5), colour difference ΔE* (vs. untreated), and CIE whiteness index (W*_CIE_*, D65/10°) for cotton (CO) and polyester (PES) fabrics: untreated (UT), SiO_2_/PDMS-finished (Si) before and after 1, 3, and 5 washes, and fluoropolymer-finished (F) before and after 1, 3, and 5 washes.

Samples	L*	a*	b*	ΔE*	W*_CIE_*
CO_UT	95.72 ± 0.06	3.20 ± 0.04	−10.65 ± 0.10	N/A ^1^	125.40 ± 1.57
CO_Si	95.34 ± 0.06	2.64 ± 0.01	−8.56 ± 0.06	2.29 ± 0.05	108.85 ± 1.29
CO_Si_1W	95.89 ± 0.02	2.92 ± 0.02	−9.92 ± 0.04	1.50 ± 0.04	114.93 ± 1.09
CO_Si_3W	95.90 ± 0.03	2.99 ± 0.01	−10.57 ± 0.05	2.12 ± 0.04	119.62 ± 0.46
CO_Si_5W	95.78 ± 0.06	2.95 ± 0.01	−10.42 ± 0.05	1.94 ± 0.03	119.08 ± 0.05
CO_F	95.77 ± 0.16	3.13 ± 0.05	−10.30 ± 0.20	0.50 ± 0.18	117.48 ± 5.29
CO_F_1W	95.91 ± 0.12	3.29 ± 0.05	−11.22 ± 0.10	0.99 ± 0.17	125.16 ± 0.33
CO_F_3W	96.03± 0.01	3.38 ± 0.01	−12.01 ± 0.05	1.79 ± 0.05	126.95 ± 0.53
CO_F_5W	95.78 ± 0.11	3.29 ± 0.03	−11.76 ± 0.01	1.51± 0.01	129.35 ± 0.12
PES_UT	94.71± 0.05	−0.64 ± 0.01	1.75 ± 0.02	N/A ^1^	76.94 ± 0.15
PES_Si	94.45 ± 0.01	−0.85 ± 0.01	3.02 ± 0.03	1.32 ± 0.03	67.28 ± 0.38
PES_Si_1W	94.87 ± 0.01	−0.85 ± 0.01	2.54 ± 0.01	0.64 ± 0.01	68.07 ± 0.30
PES_Si_3W	95.11 ± 0.13	−0.82 ± 0.02	2.51 ± 0.06	0.83 ± 0.01	72.75 ± 0.76
PES_Si_5W	94.67 ± 0.05	−0.85 ± 0.02	2.97 ± 0.01	0.22 ± 0.01	66.35 ± 1.10
PES_F	94.51 ± 0.01	−0.64 ± 0.02	2.21 ± 0.01	0.51 ± 0.02	71.67 ± 0.19
PES_F_1W	94.54 ± 0.10	−1.01 ± 0.05	1.89 ± 0.07	0.49 ± 0.07	76.84 ± 0.44
PES_F_3W	94.64 ± 0.03	−0.81 ± 0.01	1.97 ± 0.02	0.32 ± 0.02	79.09 ± 0.40
PES_F_5W	94.64 ± 0.17	−0.69 ± 0.02	2.02 ± 0.03	0.24 ± 0.03	75.31 ± 0.33

^1^ N/A: not available.

**Table 2:** Add-on percentage (*A*, wt%) and weight loss by washing (*WLW*, wt%) for cotton (CO) and polyester (PES) fabrics; values are reported as mean ± SD (*n* = 5).

Sample	*A* (wt%)	*WLW* (wt%)		
		1W	3W	5W
CO_Si	1.78 ± 0.024	0.52 ± 0.009	0.71 ± 0.012	0.94 ± 0.016
CO_F	0.39 ± 0.006	0.24 ± 0.003	0.27 ± 0.004	0.39 ± 0.006
PES_Si	0.92 ± 0.009	1.70 ± 0.030	2.9 ± 0.054	3.57 ± 0.069
PES_F	0.96 ± 0.014	1.46 ± 0.026	1.50 ± 0.029	1.58 ± 0.030

**Table 3:** Hydrophobic performance of cotton (CO) and polyester (PES) fabrics: ISO 4920 spray rating and AATCC TEST METHOD 193-2007 (samples coded as follows: UT: untreated; Si: SiO_2_/PDMS-finished; F: fluoropolymer-finished; 1W, 3W, 5W: after 1, 3, 5 ISO 105-C10 wash cycles).

Sample	Spray Test ISO 4920				Water/Alcohol Repellence Test AATCC 193-2007			
CO_UT	0				0			
PES_UT	0				0			
	**UW**	**1W**	**3W**	**5W**	**UW**	**1W**	**3W**	**5W**
CO_Si	100	100	100	90	8	7	7	7
CO_F	100	100	80	70	8	8	7	7
PES_Si	100	100	100	100	8	8	7	7
PES_F	100	100	100	100	8	8	8	8

**Table 4:** Bending stiffness (*B*, cN·cm^2^) calculated from overhang length *L* according to DIN 53362 (see Section 2.6) (values are mean ± SD (*n* = 5)), and water-vapour transmission rate for cotton and polyester fabrics, both untreated and treated with sol–gel and fluorine-based finishes.

Sample	Bending Stiffness (cN·cm^2^)	Water-Vapour Transmission Rate (g·(m^2^·d)^−1^)
CO_UT	12.0 ± 0.5	905 ± 31
CO_Si	13.8 ± 0.6	869 ± 15
CO_F	15.2 ± 0.7	810 ± 21
PES_UT	9.0 ± 0.4	797 ± 28
PES_Si	10.7 ± 0.5	763 ± 17
PES_F	13.0 ± 0.6	708 ± 18

## Data Availability

The original contributions presented in this study are included in the article/Supplementary Materials. Further inquiries can be directed to the corresponding authors.

## Supplementary Materials

The following supporting information can be downloaded at: https://www.mdpi.com/article/10.3390/polym17111578/s1, Figure S1: Normalised ATR FT-IR spectra of silica-based (**a**) and fluorine-based (**b**) chemicals treated cotton fabrics compared to pristine samples, before (_UW) and after 1, 3 and 5 washing cycles (_1W, _3W_5W, respectively); Figure S2: Normalised ATR FT-IR spectra of silica-based (**a**) and fluorine-based (**b**) chemicals treated polyester fabrics compared to pristine samples, before (_UW) and after 1, 3 and 5 washing cycles (_1W, _3W, _5W, respectively); Figure S3: Natural-log plot of hydrophobic performance versus laundering cycles. The ordinate shows ln (θ_n_/θ_0_), where θ_0_ is the static contact angle before washing and θ_n_ the angle after *n* ISO 105-C10 cycles (*n* = 1, 3, 5). Lines are least-squares fits drawn through the four experimental points (0, 1, 3, 5) for each finish: SiO_2_/PDMS on cotton (CO_Si), SiO_2_/PDMS on polyester (PES_Si), fluoropolymer on cotton (CO_F) and fluoropolymer on polyester (PES_F). The negative slopes give the apparent wash-off rate constants k (wash^−1^).
