# Supplementary material for: Eco-Friendly Octylsilane-Modified Amino-Functional Silicone Coatings for a Durable Hybrid Organic–Inorganic Water-Repellent Textile Finish
Source: Polymers (Basel). 2025 Jun 5;17(11):1578. doi: 10.3390/polym17111578 (PMC12157011; doi:10.3390/polym17111578)
Supplement: Supplementary file 1 [file polymers-17-01578-s001.zip › polymers-3644305-supplementary.pdf]

Supplementary Information

Eco-Friendly Octylsilane-Modified Amino-Functional Silicone Coatings for a Durable Hybrid Organic–Inorganic Water-Repellent Textile Finish

Mariam Hadhri[1], Claudio Colleoni[2], Agnese D'Agostino[1,*], Mohamed Erhaim[1], Raphael Palucci Rosa[1], Giuseppe Rosace[1,3,4], Valentina Trovato[1,3*]

[1] Department of Engineering and Applied Sciences, University of Bergamo, 24044 Dalmine, BG, Italy; mariam.hadhri@unibg.it (M.H.); m.erhaim@studenti.unibg.it (M.E.); raphael.rosa@unibg.it (R.P.R.); giuseppe.rosace@unibg.it (G.R.)

[2] Argochem Srl, 24044 Dalmine, BG, Italy; claudio.colleoni@argochem.eu

[3] Local CSGI (Inter-University Center for Colloid and Surface Science) Research Unit, 24044 Dalmine, BG, Italy; giuseppe.rosace@unibg.it (G.R.)

[4] Local INSTM (National Consortium of Materials Science and Technology) Research Unit, 24044 Dalmine, BG, Italy; giuseppe.rosace@unibg.it (G.R.)

* Correspondence: agnese.dagostino@unibg.it (A.D.); valentina.trovato@unibg.it (V.T.)

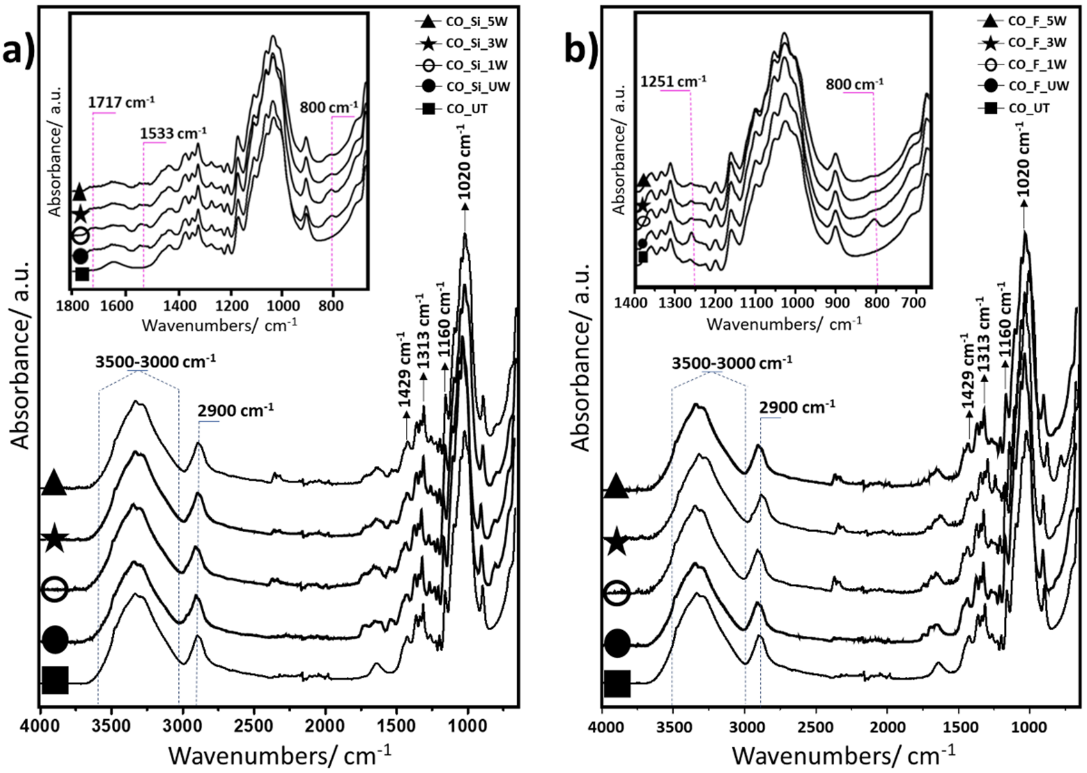

Figure S1. Normalised ATR FT-IR spectra of silica-based (**a**) and fluorine-based (**b**) chemicals treated cotton fabrics compared to pristine samples, before (_UW) and after 1, 3 and 5 washing cycles (_1W, _3W_5W, respectively).

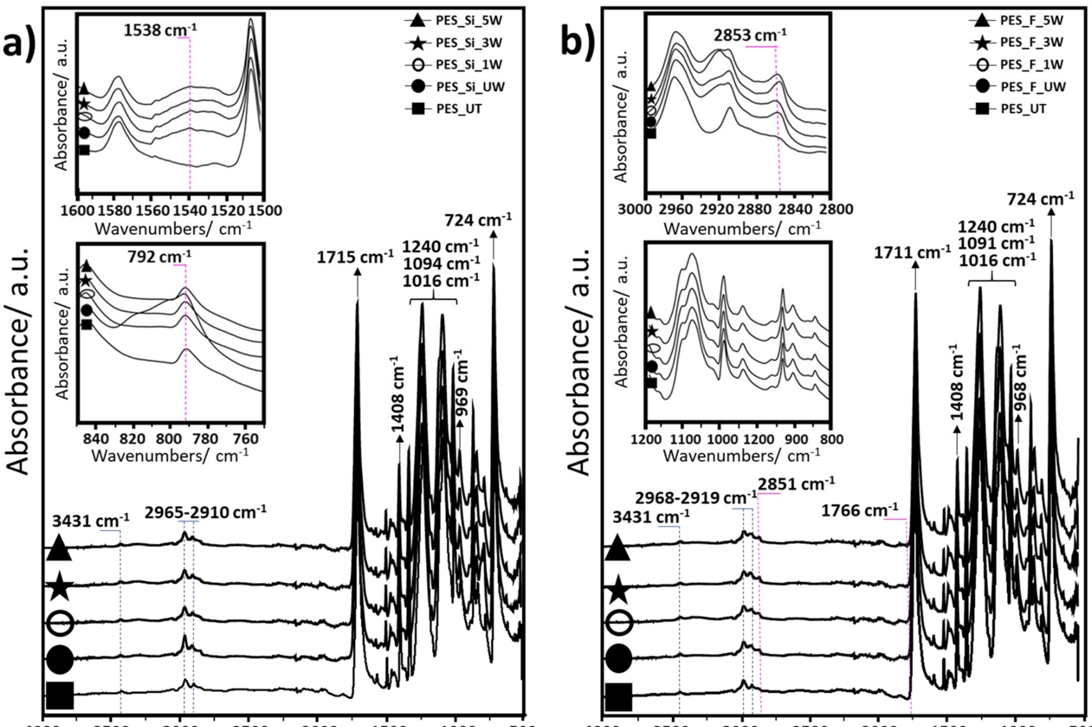

Figure S2. Normalised ATR FT-IR spectra of silica-based (**a**) and fluorine-based (**b**) chemicals treated polyester fabrics compared to pristine samples, before (_UW) and after 1, 3 and 5 washing cycles (_1W, _3W, _5W, respectively).

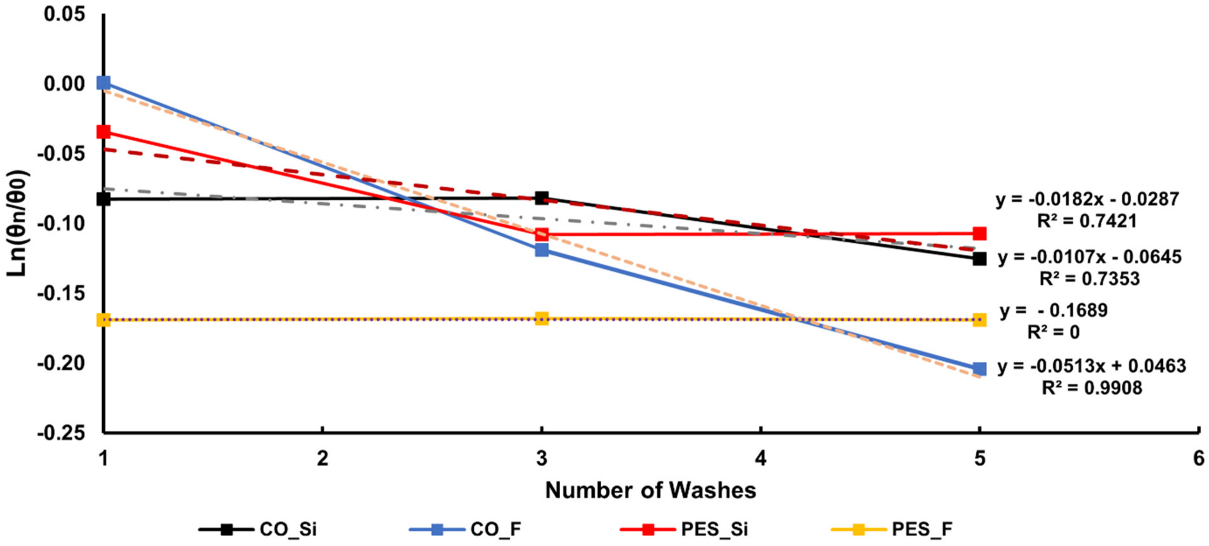

Figure S3. Natural-log plot of hydrophobic performance versus laundering cycles. The ordinate shows ln (θ_n / θ_0), where θ_0 is the static contact angle before washing and θ_n the angle after *n* ISO 105-C10 cycles (*n* = 1, 3, 5). Lines are least-squares fits drawn through the four experimental points (0, 1, 3, 5) for each finish: SiO_2/PDMS on cotton (CO_Si), SiO_2/PDMS on polyester (PES_Si), fluoropolymer on cotton (CO_F) and fluoropolymer on polyester (PES_F). The negative slopes give the apparent wash-off rate constants k ($wash^{-1}$).
